# Safety and preliminary efficacy of orally administered lyophilized fecal microbiota product compared with frozen product given by enema for recurrent *Clostridium difficile* infection: A randomized clinical trial

**DOI:** 10.1371/journal.pone.0205064

**Published:** 2018-11-02

**Authors:** Zhi-Dong Jiang, Robert R. Jenq, Nadim J. Ajami, Joseph F. Petrosino, Ashley A. Alexander, Shi Ke, Tehseen Iqbal, Andrew W. DuPont, Kenneth Muldrew, Yushu Shi, Christine Peterson, Kim-Anh Do, Herbert L. DuPont

**Affiliations:** 1 University of Texas School of Public Health, Houston, TX, United States of America; 2 MD Anderson Cancer Center, Houston, TX, United States of America; 3 Alkek Center for Metagenomics and Microbiome Research, Department of Molecular Virology and Microbiology, Baylor College of Medicine, Houston, TX, United States of America; 4 Kelsey Research Foundation, Houston, TX, United States of America; 5 University of Texas McGovern Medical School, Houston, TX, United States of America; 6 CHI St. Luke’s Health-Baylor St. Luke’s Medical Center, Houston, TX, United States of America; University Hospital Llandough, UNITED KINGDOM

## Abstract

**Background:**

Fecal microbiota transplantation (FMT) via colonoscopy or enema has become a commonly used treatment of recurrent *C*. *difficile* infection (CDI).

**Aims:**

To compare the safety and preliminary efficacy of orally administered lyophilized microbiota product compared with frozen product by enema.

**Methods:**

In a single center, adults with ≥ 3 episodes of recurrent CDI were randomized to receive encapsulated lyophilized fecal microbiota from 100–200 g of donor feces (n = 31) or frozen FMT from 100 g of donor feces (n = 34) by enema. Safety during the three months post FMT was the primary study objective. Prevention of CDI recurrence during the 60 days after FMT was a secondary objective. Fecal microbiome changes were examined in first 39 subjects studied.

**Results:**

Adverse experiences were commonly seen in equal frequency in both groups and did not appear to relate to the route of delivery of FMT. CDI recurrence was prevented in 26 of 31 (84%) subjects randomized to capsules and in 30 of 34 (88%) receiving FMT by enema (p = 0.76). Both products normalized fecal microbiota diversity while the lyophilized orally administered product was less effective in repleting Bacteroidia and Verrucomicrobia classes compared to frozen product via enema.

**Conclusions:**

The route of delivery, oral or rectal, did not influence adverse experiences in FMT. In preliminary evaluation, both routes appeared to show equivalent efficacy, although the dose may need to be higher for lyophilized product. Spore-forming bacteria appear to be the most important engrafting organisms in FMT by the oral route using lyophilized product.

**Trial registration:**

ClinicalTrials.gov NCT02449174

## Introduction

Fecal microbiota transplantation (FMT) has become widely used for the treatment of patients with ≥3 bouts of *Clostridium difficile* infection (CDI) [1] with durable response for at least 90 days [2]. In most current settings, fresh or frozen fecal microbiota product have shown equal efficacy in the treatment of recurrent CDI. Frozen product has become preferred where available to fresh product because of advantages of convenience [3–5].

Retention enema has become widely used as a route of administration of FMT and is effective [5]. In preliminary studies, the oral route has been successful for both frozen FMT product [6] and lyophilized (freeze-dried) product [7].

The present study follows our previous evaluation of fresh, frozen and lyophilized FMT products in recurrent CDI delivered by colonoscopy [8] and examines the safety and preliminary efficacy and engraftment of the microbiome when lyophilized fecal microbiota product was given orally compared with frozen product given by enema in a randomized clinical trial. We also profiled the fraction of culturable bacteria in the finished lyophilized and frozen product and compared it with the fresh donor stools to determine reduction in bacterial counts by lyophilization and freezing.

## Material and methods

### Study design

This randomized, single-center trial was carried out in a medical clinic in Houston. Randomization employed 1:1 allocation in blocks of four, using R Statistical Program (version 3.4.1, www.r-project.org). The study was blinded only for personnel performing the analyses. A data safety monitoring board (DSMB) met before the study began, after the first 15 subjects were enrolled and at study completion. The randomization list was made by the laboratory director at UTHSC (ZDJ).

*C*. *difficile* infection (CDI) was defined as the passage of ≥3 watery stools per 24 hours for at least two consecutive days, with a positive test for fecal *C*. *difficile* toxin(s) with receipt of anti-CDI antibiotics [9]. The principal study outcome was safety at three months following FMT. While not powered for these endpoints, an assessment of preliminary efficacy and microbiome restoration were performed.

#### FMT recipients

Patients with recurrent CDI presenting to our center were screened for possible enrollment. Inclusion criteria were: age ≥ 18 years; non-pregnant; ≥ 3 total episodes of CDI; receipt of at least one course of anti-CDI antibiotics for most recent bout; availability of a non-study physician for non-transplant care; ability and willingness to comply with study requirements. Study exclusions were: history of total colectomy; history of incontinence; planned receipt of concomitant antibiotics or probiotic; presence of a definable non-CDI diarrhea pathogen; known white blood count >15×10^9^/L or absolute neutrophil count < 0.5×10^9^/L; presence of toxic megacolon; history of intestinal perforation; or presence of unstable medical conditions.

#### Donor selection

Three donors selected for the study were screened for safety (history, blood tests and fecal pathogen testing) as described in a previous publication of ours [8].

#### Donors stool preparation at UTSPH-EPDL

After stool collection in a clean, closed container, 100 grams of stool/donor donation were processed within four hours of passage by mixing a 1:5 dilution in 500mL of sterile 0.85% NaCl containing a cryoprotectant followed by filtering twice through double-layered woven gauze (Fisher HealthCare, www.fishersci.com). After centrifuging at 1000xg for 10 minutes, the pellet was stored at -80°C for use as frozen FMT product. For the lyophilized product, the filtered solution was centrifuged at 2700xg for 15 minutes twice with lyophilization of the combined pellets followed by storage for approximately 4 hours at -80°C. The dried powder (averaging 1.5 g/100 g of fecal product) was then transported in sealed 50 mL conical vials on wet ice to a nearby compounding pharmacy for encapsulation using 00-size Acid Resistant capsules (Farmacapsules, Barranquilla, Colombia) and subsequently stored at standard refrigerator at 4°C. The pharmacist was not required to completely fill the capsules.

#### Fecal microbiota transplantation procedure

Enrolled subjects stopped antibiotics or probiotics 48 hours before the procedure, followed a clear liquid diet a day before FMT, ingested 10 oz. of magnesium citrate or Golyetly for subjects with kidney function impairment the night before the procedure, and took loperamide (4 mg) on the morning of the procedure. All enrolled subjects were treated and then followed at a single well supervised medical clinic.

Initially subjects randomized to oral product swallowed capsules containing 100 g of fecal microbiota under supervision and were asked to comment on their experience with swallowing of the capsules. After randomizing the first 14 subjects, 3 of 8 (38%) taking the oral product had failed treatment by developing CDI during two months post-FMT compared with 0 of 6 subjects randomized to the frozen product. With involvement of the study DSMB, subsequent subjects randomized to oral product received two doses of FMT product, each derived from 100 g of fecal microbiota, with one does in the clinic and a second equal dose of product taken at home by self-administration 24 hours later (total dose 200 g of original stool).

For subjects enrolled for frozen product FMT, the frozen product (500mL containing 100 g of fecal microbiota) was thawed at 4°C and instilled via enema and retained for 60 minutes.

#### Assessment for safety of FMT administered by mouth compared with rectal instillation

After FMT, subjects were followed for three months using subject diaries and phone calls. At each follow-up (1, 7, 14, 30, 60 and 90 days after FMT), subjects submitted a diary where they had recorded bowel movements, adverse experiences, medication, enteric symptoms, including nausea, vomiting, flatulence, abdominal cramps/pain, and urgency and other adverse experiences. An adverse experience was defined as any unfavorable or unintended sign, symptom or disease temporally associated with FMT procedure whether or not considered related to the procedure. Intensity of adverse experiences was classified as mild, moderate or severe. Subjects with unanticipated adverse experiences were contacted by the investigators to further assess events. Episodes of CDI were sought by diary and phone calls. In a preliminary evaluation of efficacy, clinical cure was defined as no episodes of CDI during the 60 days after FMT treatment.

#### Stool sample collection and sequencing

Stool samples were collected 1–2 days before FMT and then after FMT at 2 days, 7 days, 14 days, 30 days, 60 days and 90 days. If recipients developed CDI within 60 days of treatment, they were removed from further study. The initial collected stool sample pre-FMT was tested for presence of *C*. *difficile* toxin A/B by commercial enzyme immunoassay (Remel, Lenexa, KS).

#### Sequencing

Fecal microbiome analyses were performed on the first 39 subjects studied. Stool collected before FMT and 2, 30 and 90 days after FMT were included in this study. DNA isolation and microbiome sequencing were conducted at the Baylor College of Medicine’s Alkek Center for Metagenomics and Microbiome Research [10, 11]. Briefly, genomic bacterial DNA was extracted from fecal samples using the MO BIO PowerSoil DNA Isolation Kit (MO BIO Laboratories, Inc., Carlsbad, CA). One aliquot of each frozen stool sample was thawed, and 500μL of stool was transferred to a MO BIO PowerSoil DNA Extraction PowerBead Tube. Samples were incubated at 95°C for 10 minutes, then at 65°C for 10 minutes, followed by genomic DNA extraction using the MO BIO PowerSoil DNA Extraction Kit protocol. DNA samples were stored at -20°C. Genomic 16S ribosomal-RNA V4 variable region was amplified and sequenced on the Illumina MiSeq platform as previously described [12].

#### Viability of cultural anaerobic microbiota after processing

Stool samples collected from the donors used in this trial as well as a fourth donor used in other studies were evaluated for culturable anaerobic bacteria comparing fresh, frozen or lyophilized products. The fresh fecal product was filtered within 2 hours of collection and an aliquot saved. The non-saved portion was processed as described above to obtain frozen or lyophilized material. The three forms of processed fecal products were serially diluted from 1:10^3^ to 1:10^8^ in 0.85% NaCl, with 100μL of diluted fecal material plated onto blood agar before incubation at 37°C for 24 hours under anaerobic conditions for later colony counting. The number of colonies was multiplied by 6, which was the dilution factor to calculate colony-forming unit (CFU)/mL [13].

#### Microbial community composition and diversity

VSEARCH was used for analyzing nucleotide sequences [14]. Paired-end reads were merged, de-replicated, and sorted by length and size. Sequences were then error-corrected and chimera-filtered using the UNOISE algorithm (http://www.biorxiv.org/content/early/2016/10/15/081257) to generate a preliminary list of operational taxonomic units (OTUs). Both OTUs and presumed chimeras were assigned taxonomy in QIIME [15] using the Mothur method [16] with the Silva database, version 128 http://databasecommons.org/database.jsp?db_id=238. Additionally, chimeras that matched a database entry with a perfect score were restored to generate the final list of OTUs. An OTU table was generated using VSEARCH, and UniFrac [17] distances between samples were determined with QIIME. For assessment of the inverse Simpson diversity score, sample sequences were first rarefied at a number below the sample with the least number of sequences (20,000) using QIIME. In addition, principal coordinate analysis was used to illustrate the unweighted UniFrac distance between study participants and donor samples in two dimensions. PC1 and PC2 construct an orthogonal coordinate that displays the most variation between samples in two dimensions.

### Statistical analyses

Continuous variables were analyzed using a two-sided t-test and non-parametric Mann-Whitney test, as appropriate. The chi-square test was used to compare categorical variables.

In developing a sample size in this study of safety, we wanted to include the approximate number of subjects as we did in our previous trial comparing frozen or lyophilized product with fresh product given by colonoscopy where 73 subjects were enrolled providing for approximately 25 successfully studied subjects per group completing the trial [8]. We planned to enroll 65 subjects for the two groups to have sufficient power to compare safety. This approach was approved by the study DSMB.

## Results

### Subjects enrolled

**Fig 1:**
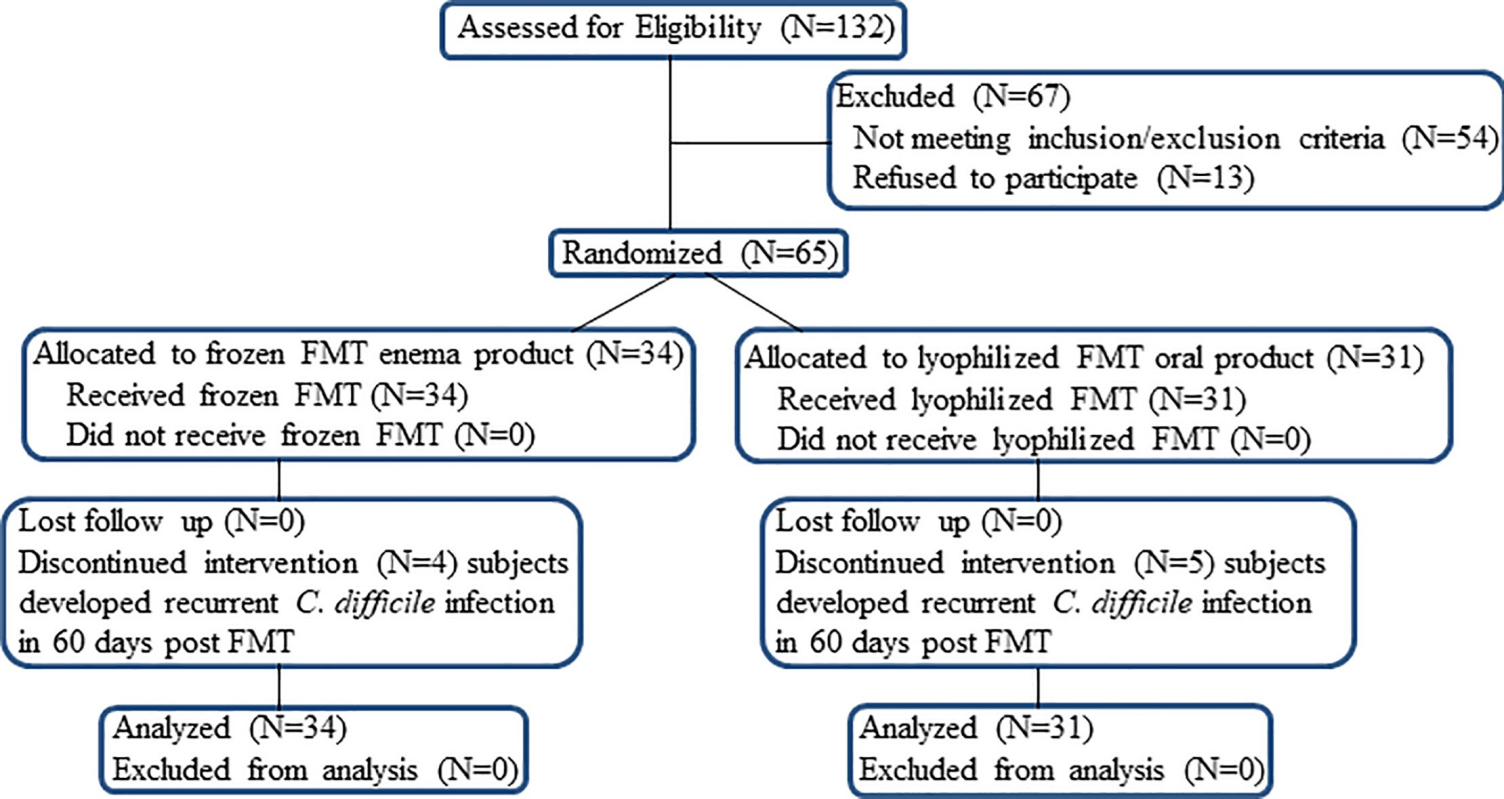
Subject evalation and disposition.

One hundred thirty-two patients with a history of recurrent CDI were evaluated for inclusion in the study (Fig 1). Sixty-seven subjects were excluded based on exclusion and inclusion criteria. Four subjects receiving frozen product and five subjects receiving lyophilized product were censored during the two months after FMT because of CDI development. FMT was performed on 65 subjects meeting our criteria at a single center between March 1, 2015 and July 31, 2017.

Subjects’ demographics and presence or absence of pre-existing inflammatory bowel disease (IBD) are listed in Table 1. Thirty-one subjects received FMT via oral capsules and 34 subjects received FMT by enema. Females predominated among the subjects. The mean duration from the last episode of CDI before study FMT was 1.8 months, ranging from 0.6 to 7 months. The average number of capsules provided was 27 per subject in that study arm. None of the subjects randomized to capsules indicated that the taste or smell was objectionable, and all were able to swallow and retain the capsules.

**Table 1:** Characteristic of subjects with recurrent CDI at enrollment for FMT.

Group	N	Age (years)			Female	Pre-Existing IBD	Number of CDI Episodes Pre-FMT (Mean, Median, Range)	Months Since Last Episode of CDI (Mean, Median, Range)
		Mean	Median	Range				
Frozen FMT	34	63	71	20–95	25 (74%)	5 (15%)	3.9, 4, 3–7	1.8, 1.6, 0.7–7
Lyophilized FMT	31	67	71	28–97	21 (68%)	6 (19%)	3.9, 4, 3–6	1.7, 1.5, 0.6–4.9

IBD = pre-existing inflammatory bowel disease

### Safety

A summary of adverse experiences (AEs) can be seen in Table 2 looking at the week after the FMT, as well as for the time period of three-month after treatment. We found the AE profile in the two groups receiving oral lyophilized product to be similar, so these groups were combined. No significant differences were observed for any gastrointestinal symptoms between lyophilized and frozen groups during the 7 days after treatment, although there was a higher percent of subjects randomized to receive oral product who experienced nausea or fecal urgency. A higher percentage of subjects receiving FMT by the rectal route experienced abdominal cramps. We saw no differences between the two groups for the 3 months after FMT. Many of the complaints occurring after 7 days appeared to be related to underlying medical conditions including inflammatory bowel disease, urinary tract infection, or other defined disorder.

**Table 2:** Subject-reported Adverse Experiences (AEs) by treatment group.

Category	AEs During First 7 Days after FMT			Total AEs for the 3 Months-Time Period after FMT		
	Combined Lyophilized n (%)	Frozen Enema n (%)	*P value*	Combined Lyophilized n (%)	Frozen Enema n (%)	*P value*
Total Subjects (N)	31	34		31	34	
Diarrhea	12(39)	10(29)	*0*.*28*	14 (45)	16 (47)	*1*.*00*
Nausea	13(42)	7(21)	*0*.*12*	15 (48)	12 (35)	*0*.*32*
Vomiting	2(7)	2(6)	*1*.*00*	7 (22)	2 (6)	*0*.*07*
Abdominal cramps/pain	17(55)	24(71)	*0*.*21*	21 (68)	26(76)	*0*.*78*
Flatulence	8(26)	11(32)	*0*.*60*	13 (42)	15 (44)	*1*.*00*
Fecal Urgency	14(45)	10(29)	*0*.*21*	22 (71)	21 (62)	*0*.*60*
Constipation	1(3)	3(8)	*0*.*62*	4 (13)	6 (18)	*0*.*74*
Other AEs	10(32)	8(24)	*0*.*58*	15 (44)	18 (53)	*0*.*81*

Nine serious adverse experiences were reported in the study including 4 of 31 (13%) in the group receiving lyophilized product orally and 5 of 34 (15%) in the group receiving frozen product by enema group (p = 1.00). In the group receiving lyophilized product, serious adverse experiences included: two hospitalizations (one for CDI recurrence two days after FMT and one for of pneumonia, fourteen days after FMT), and two deaths (one cerebral vascular accident fourteen days after FMT and one COPD/cardiac failure five months after FMT). In the group receiving frozen product by enema, serious adverse experiences included four hospitalizations: one due to IBD flare up seven days after FMT; two from CDI recurrences, one at seven days and one at fourteen days after FMT; diverticulitis, five months after FMT; and one death due to brain concussion from a fall three-months after FMT. None of the serious adverse events were felt to be related to the FMT. All three deceased subjects in the study had significant baseline comorbidities and none suffered from CDI at time of death.

### Preliminary assessment of efficacy

Overall 56 of 65 (86%) subjects enrolled to the study were cured with no episodes of CDI during the two months after FMT (Table 3). Five of the first 8 (63%) subjects randomized to receive lyophilized product in a single oral dose of FMT derived from 100 g of stool were cured. In the subgroup receiving two doses of oral FMT (total 200 g fecal product), 21 of 23 (91%) subjects were cured (p = 0.61) with an overall cure rate by oral capsule form of FMT of 26 of 31 (84%). In the group randomized to receive frozen FMT via enema, 30 of 34 (88%) were cured (p = 0.73 for oral versus frozen).

**Table 3:** Preliminary efficacy by treatment group in preventing CDI recurrence for 60 days after FMT.

Treatment Group	Lyophilized* 100 g FMT product once	Lyophilized* 100 g FMT product for 2 consecutive days (total 200 g)	Total Lyophilized* groups combined	Frozen+ 100 g FMT product once
Number Subjects	8	23	31	34
Cure* (%)	5 (63%)	21 (91%)	26 (84%)	30 (88%)

*Lyophilized product given orally in enteric coated capsules

+Frozen product given by retention enema

p values

Overall comparison: Lyophilized (total 31) vs. Frozen (total 34), p = 0.76 with 95%CI -2.86 to 0.78

High dose lyophilized (total 23) vs. Frozen (total 34), p = 0.54 with 95%CI -4.26 to 0.12

Low Dose lyophilized (total 8) vs. Frozen (total 34), p = 0.11 with 95%CI -2.86 to 0.78

Low Dose lyophilized (total 8) vs. High dose lyophilized (total 23), p = 0.09 with 95%CI -1.22 to 0.02

The subject cure rate seen for product from the three donors were similar 28 of 33 (85%), 22 of 25 (88%), and 6 of 7 (86%) (p = 0.12).

### Presence of fecal *C*. *difficile* toxin at the time of FMT

Stools from 6 of the 56 subjects (11%) were positive for *C*. *difficile* toxin(s) by EIA just before receipt of FMT. Of the 9 subjects developing CDI during the two months after FMT, 1 (11%) was previously positive for toxin in the pre-FMT sample compared with 5 of 56 (9%) of the subjects not experiencing CDI after FMT (p = 0.6069).

### Reduction in viable bacteria by lyophilization or freezing

Quantitative counts of culturable anaerobic bacteria could be performed at a 1:10^6^ dilution of donor stools, yielding 495.5±354.7 colonies for fresh, 136.8±140.4 for frozen and 290.6±207.0 for lyophilized FMT products (p = 0.014). The viable/cultural anaerobic colony count was significantly lower in the frozen (p = 0.015) and lyophilized fecal product (p = 0.029) compared with the fresh fecal material.

### Microbiota characterization

**Fig 2:**
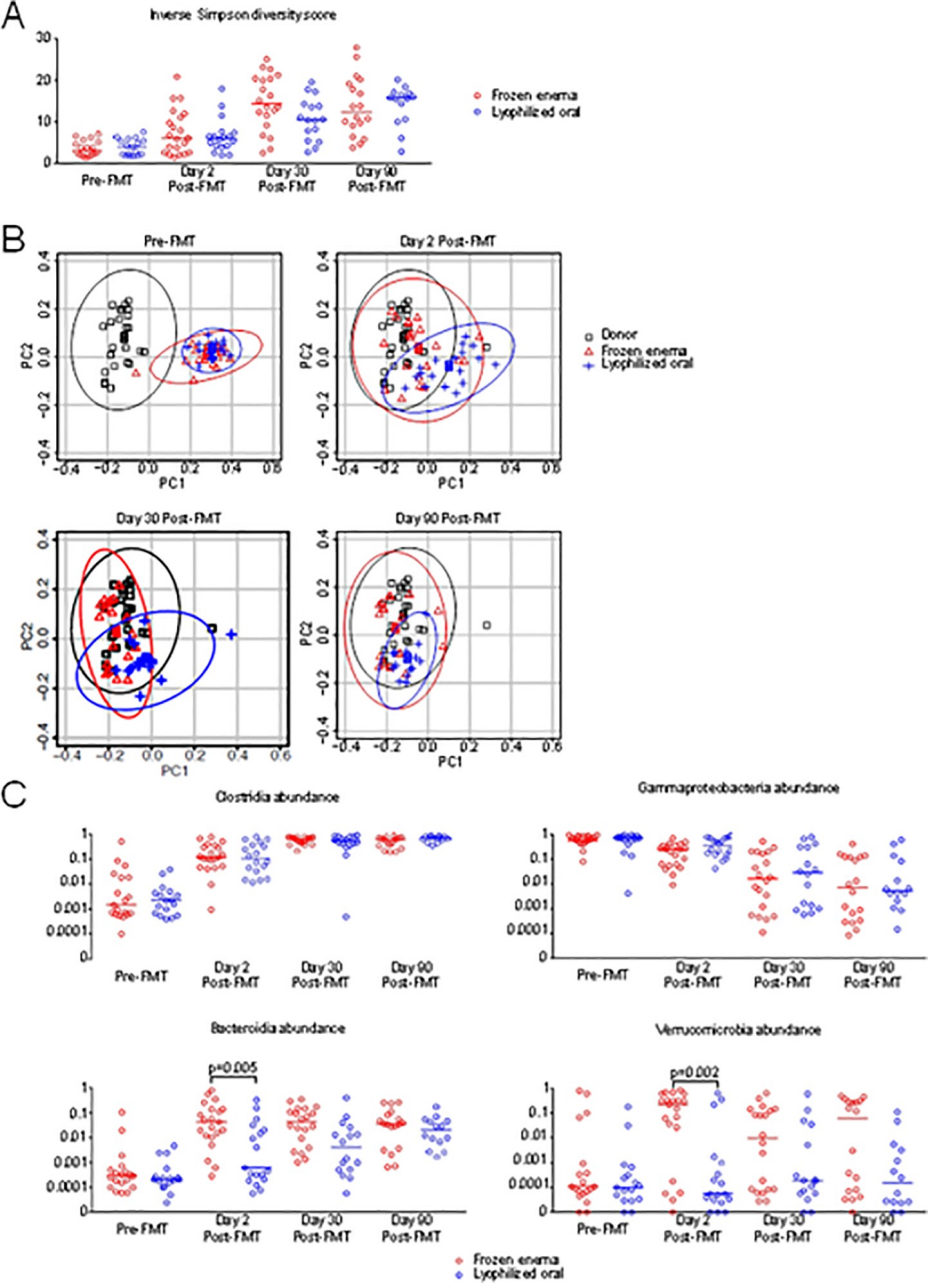
Microbiota analysis of 39 subjects before and after receiving FMT. A) Microbial diversity, quantified by the inverse Simpson index which combines measures of richness and evenness, of subject samples collected pre-FMT and post-FMT on the indicated time points. B) Principal Coordinate Analysis (PCoA) of UniFrac distances at specified time points, as well as donor samples. The axis PC1 explains 20.03% variation of the data and PC2 explains 6.13% variation of the data. Ellipses depict 95% confidence regions for each group. C) Bacterial abundance at the Class level of taxonomy of subject samples at indicated time points.

Microbial diversity, as measured by the inverse Simpson index, showed low baseline diversity in the 39 subjects with CDI pre-FMT, with minor improvement by day 2, after transplantation, near complete recovery as previously defined [11] by day 30 (Fig 2A), with no differences between the two study groups. However, differences in the pace of recovery in the two treatment groups could be appreciated by comparing the subject samples with the FMT donor samples using UniFrac distance [17], which takes into account relatedness between operational taxonomic units (OTUs). At baseline prior to FMT, both treatment groups were predominately distinct in their microbiome profile from donor samples. As early as 2 days following FMT, however, this difference was reduced with both modes of FMT administration and was further reduced at later time points (Fig 2B). Notably, recipients of frozen product by enema on Day 2 had more rapidly changed to resemble their FMT donors (median Day 2 UniFrac distance 0.51 in recipients of frozen product by enema vs 0.59 in recipients of oral lyophilized product, p = 0.0003), indicating frozen enema was more effective at normalizing the microbiome at this early stage. These changes became less apparent with later time points after FMT.

Examining the major subsets of intestinal bacteria at the Class taxonomic level (Fig 2C), we found that Clostridia were efficiently transferred by both treatment modalities. Corresponding to increasing engraftment of Clostridia, Gammaproteobacteria levels were reduced in a similar manner with both treatment types. However, two major classes of intestinal bacteria, Bacteroidia and Verrucomicrobia, were inefficiently acquired by recipients receiving lyophilized oral FMT in contrast to their efficient transfer with frozen enema FMT. In the FMT recipients by day 90, abundance of Bacteroidia were equivalent in both treatment groups while for Verrucomicrobia, the group receiving lyophilized material did not reach the abundance levels seen for the group receiving frozen product (Fig 2).

## Discussion

We have previously shown that freeze-drying fecal microbiota, without a cryoprotectant provided somewhat reduced efficacy compared with fresh microbiota, with frozen product in between, when administered by colonoscopy to subjects with recurrent CDI [8].

In the present study, we wanted to determine relative safety of FMT product given orally compared with enema delivery. While the frequency of nausea was higher in subjects receiving orally administered capsules, this rate was not significantly different than that seen in the group receiving rectal instillation and rates of vomiting were low and similar in the two groups. Overall the microbiota products were safely administered with no apparent difference between orally administered lyophilized FMT versus frozen product given by enema.

In the present study, we used a cryoprotectant in the preparation of both lyophilized and frozen products which appeared to be associated with improved protection. We found that lyophilized orally administered FMT product in enteric-coated capsules was as effective in producing CDI cure as frozen product given by enema when we administered the oral product in twice the dose and given on two consecutive days.

A recent randomized open-label FMT study of 116 patients with recurrent CDI demonstrated success of delivery by oral capsules containing frozen fecal microbiota [18]. The study showed that frozen FMT product administered in oral capsules was not inferior to delivery of the product by colonoscopy in preventing recurrent CDI during three months after the procedure. The subjects in the latter study were given 40 FMT capsules containing frozen product with 34% of subjects complaining of objectionable taste. We have identified three advantages of our lyophilized product over frozen FMT product administered in capsules assuming equal efficacy. First, the volume of product needed when delivering lyophilized product compared with frozen product would be reduced leading to fewer capsules taken. The compounding pharmacist encapsulating our product indicated that the 1.5 g of lyophilized powder derived from 100 g of stool could be put into ten capsules if packed full. Secondly, lyophilized powder should have less objectionable smell or taste than frozen product. Thirdly, lyophilized product is more easily stored at standard refrigerator temperature versus ultralow temperatures for frozen product. We also believe that enteric coating of the capsules used in this study would allow delivery into the small bowel rather than the stomach which is advantageous in case of vomiting or gastroesophageal reflux.

Presence or absence of fecal *C*. *difficile* toxins at the time of FMT had no predictive value in determining which subjects would or would not experience clinical failure after FMT. We used the EIA test which while specific is not sensitive to low levels of toxin.

Both lyophilized, orally administered and frozen enema-delivered products in the present study were similar in their ability to improve overall intestinal microbiome diversity in this study. Lyophilization appears to be harsher than freezing, leading to a reduction of certain taxa. Clostridia were preserved by both treatments, presumably because of spore formation of many of the species. Both treatments led to equivalent reduction in Gammaproteobacteria. The frozen product led to rapid engraftment of Bacteroidia class in contrast to the group receiving lyophilized product; although, the changes were similar after 90 days. The most dramatic differences were for Verrucomicrobia where efficient engraftment occurred in the group receiving frozen product, but levels remained low throughout the study for the group receiving lyophilized product orally. We postulate that these differences relate to the damaging effects of gastric acid, bile salts and digestive enzymes during the upper gastrointestinal transit for the orally administered FMT product. In our previous study [8], we observed abundance of Bacteroidia and Verrucomicrobia were comparable in lyophilized versus frozen FMT donor products given by colonoscopy at 7 days post FMT. Bacteroidia, Akkermansia and Verrucomicrobia do not form spores, in contrast to the vast majority of Clostridia. Since the oral lyophilized product appeared to be as effective as frozen product by enema, it supports the idea spore-forming Clostridia are the major protective microbial group in FMT [19]. Also, there is indirect evidence that Verrucomicrobia reconstitution is not important in recovery from recurrent CDI during FMT treatment since levels of this group never normalized in the group receiving lyophilized product in the present study. It may be, however, that certain species of Verrucomicrobia that are engrafted are more important than others complicating this conclusion. It appears that engraftment of critical taxa is key to successful FMT treatment in recurrent CDI rather than major replacement of microbiota [20].

Further evidence of the damaging effects of freezing is seen with the growth studies performed. In this study, freezing led to a 72% reduction in culturable anaerobic bacteria and freeze-drying resulted in a 41% reduction in culturable organisms. While culturable bacteria are not likely the important bacteria in the engraftment process of FMT, this study shows the damaging effect on bacteria of freezing and freeze-drying, even with a cryoprotectant. We provide preliminary data that by increasing the dose of lyophilized product and giving it on two sequential days we overcame the loss of microbiota seen by freeze drying.

Regarding limitations, with a primary interest in safety and with limited finances, we were not able to compare multiple doses employed for oral administration. The study would have been improved by increasing the sample size to allow us to randomize subjects to three groups, the two doses of lyophilized product used compared with frozen product given by enema. In addition, while we assume that both procedures preserved spore-forming *Clostridia*, no spore counts were performed in this study.

Duration of storage and stability of frozen and lyophilized FMT products is an important consideration for an FMT program. In a previous study, fecal aliquots were frozen in 10% glycerol resulting in constant number of viable and culturable bacteria for 2 to 6 months (3). Working with the frozen and lyophilized product used in the present study, we found both products active in preventing CDI in a mouse model up to 7 months after storage of, frozen product at -80^o^ C and lyophilized product at standard refrigerator temperature (~1.6^o^ C) [21].

We conclude that we have developed an effective orally administered FMT product, without odor or taste and without special storage requirements, that provides high levels of efficacy when used to prevent recurrent CDI. We are currently embarking on a dose response study to establish optimal dose of lyophilized product to use for oral administration in treatment of recurrent CDI.

## Supporting information

S1 File CONSORT 2010 checklist. (DOC)

S2 File ClinTrial.Gov release date. (PDF)

S3 File Study protocol. (DOCX)

S4 File Study data. (XLSX)
