# Supplementary material for: Safety and preliminary efficacy of orally administered lyophilized fecal microbiota product compared with frozen product given by enema for recurrent Clostridium difficile infection: A randomized clinical trial
Source: PLoS One. 2018 Nov 2;13(11):e0205064. doi: 10.1371/journal.pone.0205064 (PMC6214502; doi:10.1371/journal.pone.0205064)
Supplement: S2 File — (PDF) [file pone.0205064.s002.pdf]

ClinicalTrials.gov PRS

Protocol Registration and Results System

Contact ClinicalTrials.gov PRS

Org: UTexas_Houston User: zjiang Logout

Home > Record Summary

ID: HSC-SPH-14-0020 Fecal Microbiota Transplantation to Treat Recurrent C. Difficile Associated Diarrhea Via Retention Enema or Oral Route NCT02449174

Record Summary

Home Help

Record Status

In Progress → Entry Completed → ***Approved*** → Released → PRS Review → Public

Reset to In-Progress...

Next Step: Correct Error(s)

Record Owner:	zjiang	Access List:	[] Edit
Last Update:	06/20/2018 12:46 by zjiang	Upload:	Allowed Edit
Initial Release:	12/17/2014	PRS Review:	Review History
Last Release:	11/16/2016 Receipt (PDF)	Public Site:	Last Public Release: 11/16/2016 View on ClinicalTrials.gov
Results Expected:	No later than May 2019	FDAAA:	Probable ACT
All Results Expected:	No later than December 2018		

Spelling Preview Draft Receipt (PDF RTF) Download XML

Open **Protocol Section**

Identifiers: NCT02449174 Unique Protocol ID: HSC-SPH-14-0020

Brief Title: Fecal Microbiota Transplantation to Treat Recurrent C. Difficile Associated Diarrhea Via Retention Enema or Oral Route

Module Status:

- Study Identification: ✓
- Study Status: 4 Errors 1 Warning 1 Note
- Sponsor/Collaborators: ✓
- Oversight: ✓
- Study Description: ✓
- Conditions: ✓
- Study Design: ✓
- Arms and Interventions: ✓
- Outcome Measures: ✓ 2 Notes
