# Supplementary material for: Safety and preliminary efficacy of orally administered lyophilized fecal microbiota product compared with frozen product given by enema for recurrent Clostridium difficile infection: A randomized clinical trial
Source: PLoS One. 2018 Nov 2;13(11):e0205064. doi: 10.1371/journal.pone.0205064 (PMC6214502; doi:10.1371/journal.pone.0205064)
Supplement: S3 File — (DOCX) [file pone.0205064.s003.docx]

| **Study Protocol** | |
| --- | --- |
| **Brief Title** | Fecal Microbiota Transplantation to Treat Recurrent C. Difficile Associated Diarrhea Via Retention Enema or Oral Route |
| **Official Title** | A Study of Fecal Microbiota Transplantation (FMT) for the Treatment of Recurrent C. Difficile Associated Diarrhea (RCDAD) Via Retention Enema or Oral Route |
| **Brief Summary** | The objective of the study is to investigate the safety of a frozen or lyophilized inoculum administered, respectively, by retention enema or capsules in patients with recurrent C. difficile associated diarrhea (RCDAD).  This is a single center, randomized, parallel assignment, open label safety study conducted in subjects with RCDAD. Fifty subjects will be enrolled in the study and randomized at 1:1 ratio to receive frozen filtered intestinal bacteria via retention enema or lyophilized donor intestinal bacteria. All subjects will be followed for a total of 3 years after study completion.  Donors will be enrolled and screened at the laboratory in the Center for Infectious Diseases at University of Texas School of Public Health (UT-SPH). The donors will come from a variety of places, including the UT-SPH. We will screen at least 20 donors to recruit at least 15 qualified donors.  Recipients may self-refer but must have a physician who agrees to accept care of the patient following fecal microbiota transplantation (FMT). Subjects consenting to treatment at Baylor St. Luke's Medical Center (BSLMC) and the UT-SPH must be willing to self-pay for the FMT in the amount of $1,500. There will be no insurance accepted. Subjects undergoing retention enema will be treated as outpatients at either at BSLMC, Kelsey-Seybold Clinic, or at the Memorial Hermann in the Texas Medical Center. All subjects taking capsules with lyophilized intestinal bacteria will be seen at UT-SPH. Once the procedure is completed, the recipient's care will be returned to their physician. We will screen at least 75 recipients to recruit 50 qualified recipients.  The primary endpoint is to evaluate the safety of FMT by rectal or oral routes with secondary endpoint related to efficacy prevention of RCDAD. In order to monitor any health effects for safety, participants will be contacted pre- and 7, 14, 30 days, then monthly basis for the first 90 days after FMT and quarterly till 3 years after FMT. The following procedures will be completed: review recipient diary with the recipient to ensure that the following information is recorded correctly and a fresh stool sample will be collected from recipient, tested for C. difficile toxins and an aliquot (2mL) stored at -80C for microbiome analysis. Recipients will be contacted by phone for their diarrhea status on monthly basis till 90 days after FMT, then on quarterly basis till 3 years after FMT. |
| **Detailed Description** | Clinical improvement is monitored by telephone/email and or E-mail up to 3 years after FMT. Minor clinical management of the subject with over the counter medication (e.g. loperamide, acetaminophen and Saccharomyces boulardii [probiotic]) will be performed in the case of mild diarrhea and abdominal pain.  All subjects will be followed up by phone the day after FMT to assess health status during week one. Study subjects will be monitored on approximately days 7, 14 and 30, monthly for the first 3 month and quarterly for 3 years after FMT, at which time we will review the recipient diary with the recipient to review any adverse experience or medication taken since the medical history obtained at FMT.  Investigators should determine if any adverse experience or medication need to be further studied. Donor will be contacted and tested if it is necessary. All actions will be recorded on with medical condition, dates of adverse experience and medication taken, indication for new medication taken, and total daily dose.  Recipients should be instructed at FMT to contact the Investigator if they have any questions regarding adverse experience or the appropriateness of a medication after FMT.  The following definitions of terms are guided by the International Conference of Harmonization and US Code of Federal Regulations (21 CFR 312.32).  An adverse experience is any unfavorable or unintended sign, symptom, of disease temporally associated with FMT procedure, whether or not considered related to the procedure, including, but not limited to:   - Any symptom not previously reported by the recipients (Recent Medical History) - An exacerbation of a pre-existing illness, increases in the frequency and/or severity of the signs or symptoms of CDAD (defined as positive C. difficile toxin test and enteric symptoms) - A significant increase in frequency or intensity of a pre-existing episodic event or condition - A condition first detected or diagnosed after study drug administration even through the condition may have been present before the procedure Details of all adverse experiences that occur after FMT through approximately day 90 visit will be collected as indicated above. Then the subject will be followed on quarterly basis for 3 years about their CDAD and other medical concerns   Serious adverse experience is any adverse experience that:   - Results in death - Is life threatening (at immediate risk of death from the procedure as it occurred) - Requires inpatient hospitalization (overnight stay) or prolongs a current hospitalization - Causes a persistent or significant disability/incapacity - Medical important (any event that requires medical or surgical intervention to prevent one of the outcomes listed above) The investigator will exercise medical and scientific judgment when deciding whether expeditious reporting is appropriate in other situations not strictly meeting the listed criteria above. The investigators will meet/discuss with experts in the field if there is a question of whether the adverse experience would be considered serious.   Severity - The adverse experience will be documented on the appropriate page in the Patient Diary according to the following descriptors:   - Mild: associated with no limitation of usual activities or only slight discomfort - Moderate: associated with limitation of usual activities or significant discomfort - Severe: associated with inability to carry out usual activities or very marked discomfort   Relationship - the relationship of adverse experience to FMT will be assigned by the Investigator according to the following definitions:   - Probable: a reaction that follows a reasonable temporal sequence from the procedure that follows a known or expected response pattern to the suspected procedure and that could not be reasonably explained by the known characteristics of that patient's clinical state - Possible: a reaction that follows a reasonable temporal sequence from the procedure that follows a known or expected response pattern to the procedure but could readily have been produced by a number of other factors - Unlikely: a reaction that does not follow a reasonable temporal sequence from the procedure but for which causality from FMT cannot be ruled out. - Not related: a reaction for which sufficient data exist to indicate that the etiology is unrelated to the procedure Pre-existing signs and symptoms and medical conditions Medical conditions that are present at or before the procedure that manifest with the same severity or frequency will not be recorded as adverse experience. Similarly, signs or symptoms related to a pre-existing disease will not be recorded as adverse experience unless there is an increase in the severity or frequency of the signs or symptoms. These pre-existing conditions, signs, or symptoms will be recorded on the Recent Medical History Form.   Progression of underlying conditions as an adverse experience  If the progression of the underlying condition might be reasonably anticipated given the nature and severity of the underlying condition, then the progression of the underlying condition per se will not constitute an adverse experience. However, if the progression of the underlying condition is fatal, then the progression of the underlying condition should be reported as an adverse experience.  Recording and documenting adverse experience  The Investigator must completely and promptly record each new adverse experience and serious adverse experience, even if the relationship of adverse experience to the procedure is assessed by the Investigator to be "unlikely" or "not related". In addition, the investigator must document and follow serious adverse experiences that occur from the procedure through 3 years after the FMT. The Investigator should attempt, if possible, to establish a diagnosis based on the presenting signs and symptoms. If an adverse experience meets the definition of a serious adverse experience then the Investigator must also complete the serious adverse experience, and also send any supporting source documents directly to the University of Texas Health Science Center IRB as soon as the event is discovered. At each visit, after the patient has had an opportunity to mention any problems spontaneously, the Investigator (or designee) will inquire about adverse experience by asking the standard questions listed in, such as:   - Have you had any medical problems since your last visit? - Have any medical problems present at your last visit changed, i.e., stopped, worsened, or improved? - Have you taken any new medicines, other than study drug, since your last visit? Any spontaneous adverse experience information provided by the patient will be reported. If an adverse experience has not resolved at the time of the Final Visit, the Investigator should evaluate the status of the adverse experience at the Follow-Up telephone/email contact (day 60) and update to reflect the status of the adverse experience (e.g. ongoing or resolved).   Investigator reporting of serious adverse experience All serious adverse experience must be reported to the University of Texas Health Science IRB using the serious adverse experience facsimile or email or by telephone/email as soon as the serious adverse experience is discovered, and within 24 hours after the Investigator recognizes or classifies the event as a serious adverse experience. A brief description of the event must be provided at the time of the initial serious adverse experience report. The initial serious adverse experience report should be followed up by additional information using the serious adverse experience within 48 hours. The reports should identify the patient by their unique patient number instead of names. The completed serious adverse experience Form will be used by the investigators in regulatory filings. The investigator is responsible for continuing to report to the University of Texas Health Science IRB any new or relevant follow-up information obtained concerning the serious adverse experience. The results of any additional assessments conducted must be also reported to the University of Texas Health Science IRB.  Notification of post-study serious adverse experience Investigators are not obligated to actively seek follow-up information for patients with adverse experience after the conclusion of the study (i.e., > 3 years after the FMT procedure). However, if the investigator becomes aware of an adverse experience that occurs after the patient completes and the adverse experience is considered by the Investigator to be at least possibly related to study procedure, the investigator must notify the University of Texas Health Science Center IRB. |
| **Study Type** | Interventional |
| **Study Phase** | Phase 1 Phase 2 |
| **Study Design** | Allocation: Randomized Intervention Model: Parallel Assignment Masking: Double (Participant, Investigator) Primary Purpose: Treatment |
| **Condition** | C. Difficile |
| **Intervention** | - Biological: Frozen Microbiota   Frozen Mcirobiota will be delivered via enema route.   - Biological: Lyophilized Microbiota   Lyophilized Microbiota will be delivered orally. |
| **Study Arms** | - Active Comparator: Frozen Microbiota   Donor stool (greater than 150 grams) was collected <4 hours prior to the procedure and then mixed in a homogenizer with 750mL, 1:5 dilution sterilized 0.9% NaCl in a large sterilized suction canister until a smooth consistency was reached. The suspension was filtered using a coffee filter twice. The microbiota suspension (750mL) was kept at -80C labeled with ID and expiration date which was 6 months after preparation. Intervention - Frozen Microbiota will be delivered via enema  Intervention: Biological: Frozen Microbiota   - Active Comparator: Lyophilized Microbiota   Lyophilized Microbiota_Donor stool (greater than 150 grams) was collected <4 hours prior to the procedure and then mixed in a homogenizer with 750mL, 1:5 dilution sterilized 0.9% NaCl in a large sterilized suction canister until a smooth consistency was reached. The suspension was filtered using a coffee filter twice. The microbiota suspension (750mL) was starting lyophilization process within 30 minutes after completion of stool filtration. Lyophilized microbiota products were kept at 4C and were used within 6 months after preparation. Intervention - Lyophilized Microbiota will be delivered orally  Intervention: Biological: Lyophilized Microbiota |
| **Publications *** | - [Gravel D, Gardam M, Taylor G, Miller M, Simor A, McGeer A, Hutchinson J, Moore D, Kelly S, Mulvey M; Canadian Nosocomial Infection Surveillance Program. Infection control practices related to Clostridium difficile infection in acute care hospitals in Canada. Am J Infect Control. 2009 Feb;37(1):9-14. doi: 10.1016/j.ajic.2008.07.012.](https://clinicaltrials.gov/ct2/bye/rQoPWwoRrXS9-i-wudNgpQDxudhWudNzlXNiZip9Ei7ym67VZRC5agFjSg4BA6h9Ei4L3BUgWwNG0it.) - [Dupont HL. Diagnosis and management of Clostridium difficile infection. Clin Gastroenterol Hepatol. 2013 Oct;11(10):1216-23; quiz e73. doi: 10.1016/j.cgh.2013.03.016. Epub 2013 Mar 28. Review.](https://clinicaltrials.gov/ct2/bye/rQoPWwoRrXS9-i-wudNgpQDxudhWudNzlXNiZip9Ei7ym67VZR0RLg4nFRFnA6h9Ei4L3BUgWwNG0it.) - [Jiang ZD, Hoang LN, Lasco TM, Garey KW, Dupont HL. Physician attitudes toward the use of fecal transplantation for recurrent Clostridium difficile infection in a metropolitan area. Clin Infect Dis. 2013 Apr;56(7):1059-60. doi: 10.1093/cid/cis1025. Epub 2012 Dec 7.](https://clinicaltrials.gov/ct2/bye/rQoPWwoRrXS9-i-wudNgpQDxudhWudNzlXNiZip9Ei7ym67VZR0RSR0RLK45A6h9Ei4L3BUgWwNG0it.) - [Kelly CP, LaMont JT. Clostridium difficile--more difficult than ever. N Engl J Med. 2008 Oct 30;359(18):1932-40. doi: 10.1056/NEJMra0707500. Review. Erratum in: N Engl J Med. 2010 Oct 14;363(16):1585.](https://clinicaltrials.gov/ct2/bye/rQoPWwoRrXS9-i-wudNgpQDxudhWudNzlXNiZip9Ei7ym67VZRCJxgFjWKCVA6h9Ei4L3BUgWwNG0it.) - [Chang JY, Antonopoulos DA, Kalra A, Tonelli A, Khalife WT, Schmidt TM, Young VB. Decreased diversity of the fecal Microbiome in recurrent Clostridium difficile-associated diarrhea. J Infect Dis. 2008 Feb 1;197(3):435-8. doi: 10.1086/525047.](https://clinicaltrials.gov/ct2/bye/rQoPWwoRrXS9-i-wudNgpQDxudhWudNzlXNiZip9Ei7ym67VZRCJaKC5ER05A6h9Ei4L3BUgWwNG0it.) - [Tvede M, Rask-Madsen J. Bacteriotherapy for chronic relapsing Clostridium difficile diarrhoea in six patients. Lancet. 1989 May 27;1(8648):1156-60.](https://clinicaltrials.gov/ct2/bye/xQoPWwoRrXS9-i-wudNgpQDxudhWudNzlXNiZip9Ei7ym67VZR08cg0wFg495d-3Ws8Gpw-PSB7gW.) - [Khoruts A, Dicksved J, Jansson JK, Sadowsky MJ. Changes in the composition of the human fecal microbiome after bacteriotherapy for recurrent Clostridium difficile-associated diarrhea. J Clin Gastroenterol. 2010 May-Jun;44(5):354-60. doi: 10.1097/MCG.0b013e3181c87e02.](https://clinicaltrials.gov/ct2/bye/rQoPWwoRrXS9-i-wudNgpQDxudhWudNzlXNiZip9Ei7ym67VZR0tEg4JcK4jA6h9Ei4L3BUgWwNG0it.) - [Yoon SS, Brandt LJ. Treatment of refractory/recurrent C. difficile-associated disease by donated stool transplanted via colonoscopy: a case series of 12 patients. J Clin Gastroenterol. 2010 Sep;44(8):562-6. doi: 10.1097/MCG.0b013e3181dac035.](https://clinicaltrials.gov/ct2/bye/rQoPWwoRrXS9-i-wudNgpQDxudhWudNzlXNiZip9Ei7ym67VZR0tWg0RLK4JA6h9Ei4L3BUgWwNG0it.) - [Silverman MS, Davis I, Pillai DR. Success of self-administered home fecal transplantation for chronic Clostridium difficile infection. Clin Gastroenterol Hepatol. 2010 May;8(5):471-3. doi: 10.1016/j.cgh.2010.01.007. Epub 2010 Feb 1.](https://clinicaltrials.gov/ct2/bye/rQoPWwoRrXS9-i-wudNgpQDxudhWudNzlXNiZip9Ei7ym67VZR0taRCwSg4RA6h9Ei4L3BUgWwNG0it.) |
